# 4-(1*H*-Tetra­zol-5-yl)-1*H*-indole

**DOI:** 10.1107/S1600536810033271

**Published:** 2010-08-25

**Authors:** Yu-Hua Ge, Pei Han, Ping Wei, Ping-Kai Ou-yang

**Affiliations:** a College of Life Science and Pharmaceutical Engineering, Nanjing University of Technology, Nanjing, People’s Republic of China; b Department of Chemistry and Chemical Engineering, Southeast Universiy, Nanjing 211189, People’s Republic of China

## Abstract

There are two mol­ecules with similar configurations in the asymmetric unit of the title compound, C_9_H_7_N_5_, which are linked by inter­molecular N—H⋯N hydrogen bonds into chains with graph-set motif *C*_2_^2^(8) along the *b* axis. The indole core has the expected planar geometry in the two mol­ecules, with a maximum deviation of 0.008 (8) Å from the least-squares plane defined by the nine constituent atoms, and the dihedral angles between the indole and tetra­zole rings are similar [42.4 (2) and 42.7 (2)°].

## Related literature

For the biological properties of indole and its derivatives, see: Takatoshi & Makoto (1994 ▶). For physical properties of tetra­zole, see: Itoh *et al.*, (1995 ▶). For pharmacological properties of compounds with tetra­zole and indole rings, see: Semenov (2002 ▶). For the synthesis of 5-cyano­indole, see: Frederick (1949 ▶). For hydrogen-bond motifs, see: Bernstein *et al.* (1995 ▶).
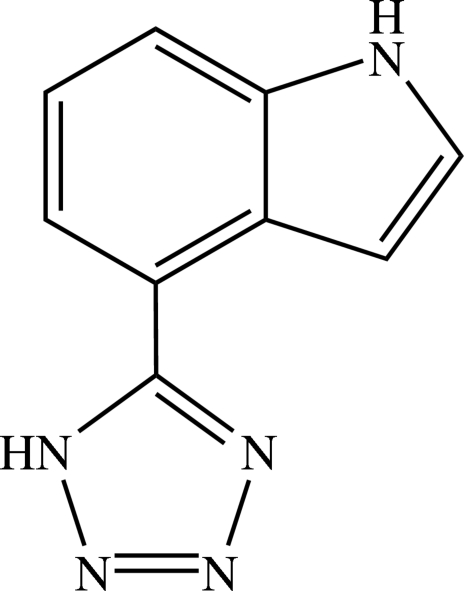


## Experimental

### 

#### Crystal data

C_9_H_7_N_5_
*M* *_r_* = 185.20
Triclinic, 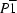

*a* = 9.6535 (7) Å
*b* = 9.8444 (4) Å
*c* = 9.9672 (7) Å
α = 83.204 (3)°
β = 65.712 (6)°
γ = 87.627 (3)°
*V* = 857.28 (9) Å^3^
*Z* = 4
Mo *K*α radiation
μ = 0.10 mm^−1^
*T* = 293 K
0.30 × 0.15 × 0.15 mm

#### Data collection

Bruker SMART 1K CCD area-detector diffractometer
Absorption correction: multi-scan (*CrystalClear*; Rigaku, 2005 ▶) *T*_min_ = 0.737, *T*_max_ = 1.000
6939 measured reflections
2990 independent reflections
2403 reflections with *I* > 2σ(*I*)
*R*_int_ = 0.037

#### Refinement

*R*[*F* ^2^ > 2σ(*F* ^2^)] = 0.076
*wR*(*F* ^2^) = 0.240
*S* = 1.02
2990 reflections
253 parameters
H-atom parameters constrained
Δρ_max_ = 0.39 e Å^−3^
Δρ_min_ = −0.30 e Å^−3^

### 

Data collection: *CrystalClear* (Rigaku, 2005 ▶); cell refinement: *CrystalClear*; data reduction: *CrystalClear*; program(s) used to solve structure: *SHELXS97* (Sheldrick, 2008 ▶); program(s) used to refine structure: *SHELXL97* (Sheldrick, 2008 ▶); molecular graphics: *SHELXTL* (Sheldrick, 2008 ▶); software used to prepare material for publication: *SHELXTL*.

## Supplementary Material

Crystal structure: contains datablocks I, global. DOI: 10.1107/S1600536810033271/bx2301sup1.cif

Structure factors: contains datablocks I. DOI: 10.1107/S1600536810033271/bx2301Isup2.hkl

Additional supplementary materials:  crystallographic information; 3D view; checkCIF report

## Figures and Tables

**Table 1:** Hydrogen-bond geometry (Å, °)

*D*—H⋯*A*	*D*—H	H⋯*A*	*D*⋯*A*	*D*—H⋯*A*
N1—H1*A*⋯N9	0.86	2.00	2.858 (4)	177
N6—H6*A*⋯N4^i^	0.86	2.10	2.899 (4)	154

Symmetry code: (i) 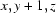.

**Fig. 1.**
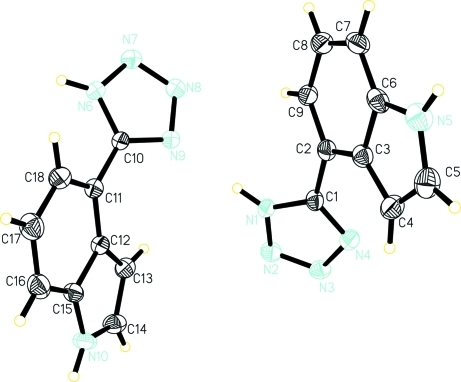
The molecular structure of the title compound, showing 30% probability displacement ellipsoids and the the atom-numbering scheme.

**Fig. 2.**
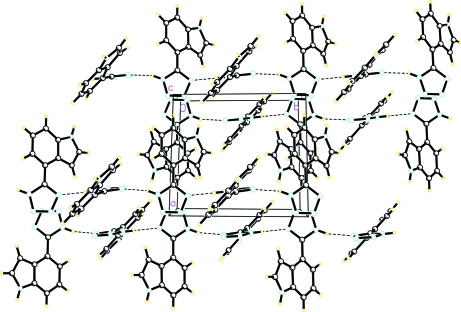
The molecular packing of the title compound (I). Hydrogen bonds are shown as dashed lines.

## supplementary crystallographic information

### Comment

Indole and its derivatives always possess good physiological activity, which are widely used as medicine, pesticide and organic chemical intermediates (Takatoshi & Makoto, 1994). Meanwhile, tetrazole is not only of good anticancer activities but also a kind of excellent ligands, it can coordinate all kinds of metal ions to form the complexes with significant optical activity (Itoh *et al.*,  1995). In recent decades, there are some reports on the compounds which are synthesized by the combination of the tetrazole and indole rings, and the property study reveals that these compounds always perform unique pharmacological activities (Semenov, 2002).We report here the compound structure of 4- (1-H-tetrazol-5-yl)-1H-indole, (I). As far as we know, there are no reports on the indole connecting the tetrazole on the C atom. In the title compound C_9_H_7_N_5_, (I) there two molecules in the asymmetric unit which are linked by one intra and intermolecular N—H···N hydrogen bond with set graph-motif C^2^_2_(8) along b axis (Bernstein *et al.*, 1995), Fig. 2. The indole core has the expected planar geometry. In both molecules of the symmetric unit the dihedral angles between the indole and tetrazole rings are very close similar 42.4 (2) and 42.7 (2)° so, the two crystallographically independent molecules have almost the same extended conformations and similar bond lengths and angles.

### Experimental

All chemicals used (reagent grade) were commercially available. 5-Cyanoindole is synthesized following the methods described by Frederick (Frederick, 1949). To the stirring DMF solution of NaN_3_ and Triethylamine, 5-Cyanoindole was added. Then the whole mixture was heated to 120°C, 1 h later, the solution was cooled to room temperature, and DMF was distilled in vacuum. With some follow-up treatment, the crude product was recrystallized in methanol and seven days later, yellow prism crystal was obtained.

### Refinement

In general, H atoms bound to carbon were placed in geometrical positions and refined using a riding model, with C—H = 0.93 and

N—H = 0.86 Å, *U*_iso_(H) =1.2*U*_eq_(C,N).

### Crystal data

**Table:**

C_9_H_7_N_5_	*Z* = 4
*M**_r_* = 185.20	*F*(000) = 384
Triclinic, *P*1	*D*_x_ = 1.435 Mg m^−^^3^
Hall symbol: -P 1	Mo *K*α radiation, λ = 0.71073 Å
*a* = 9.6535 (7) Å	Cell parameters from 2795 reflections
*b* = 9.8444 (4) Å	θ = 3.1–27.5°
*c* = 9.9672 (7) Å	µ = 0.10 mm^−^^1^
α = 83.204 (3)°	*T* = 293 K
β = 65.712 (6)°	Prism, yellow
γ = 87.627 (3)°	0.30 × 0.15 × 0.15 mm
*V* = 857.28 (9) Å^3^	

### Data collection

**Table:**

Bruker SMART 1K CCD area-detector diffractometer	2990 independent reflections
Radiation source: fine-focus sealed tube	2403 reflections with *I* > 2σ(*I*)
graphite	*R*_int_ = 0.037
Detector resolution: 13.6612 pixels mm^-1^	θ_max_ = 25.0°, θ_min_ = 3.1°
CCD_Profile_fitting scans	*h* = −11→11
Absorption correction: multi-scan (*CrystalClear*; Rigaku, 2005)	*k* = −11→11
*T*_min_ = 0.737, *T*_max_ = 1.000	*l* = −11→11
6939 measured reflections	

### Refinement

**Table:**

Refinement on *F*^2^	Primary atom site location: structure-invariant direct methods
Least-squares matrix: full	Secondary atom site location: difference Fourier map
*R*[*F*^2^ > 2σ(*F*^2^)] = 0.076	Hydrogen site location: inferred from neighbouring sites
*wR*(*F*^2^) = 0.240	H-atom parameters constrained
*S* = 1.02	*w* = 1/[σ^2^(*F*_o_^2^) + (0.1252*P*)^2^ + 1.2926*P*] where *P* = (*F*_o_^2^ + 2*F*_c_^2^)/3
2990 reflections	(Δ/σ)_max_ < 0.001
253 parameters	Δρ_max_ = 0.39 e Å^−^^3^
0 restraints	Δρ_min_ = −0.30 e Å^−^^3^

### Special details

**Table:**

Geometry. All e.s.d.'s (except the e.s.d. in the dihedral angle between two l.s. planes) are estimated using the full covariance matrix. The cell e.s.d.'s are taken into account individually in the estimation of e.s.d.'s in distances, angles and torsion angles; correlations between e.s.d.'s in cell parameters are only used when they are defined by crystal symmetry. An approximate (isotropic) treatment of cell e.s.d.'s is used for estimating e.s.d.'s involving l.s. planes.
Refinement. Refinement of *F*^2^ against ALL reflections. The weighted *R*-factor *wR* and goodness of fit *S* are based on *F*^2^, conventional *R*-factors *R* are based on *F*, with *F* set to zero for negative *F*^2^. The threshold expression of *F*^2^ > σ(*F*^2^) is used only for calculating *R*-factors(gt) *etc*. and is not relevant to the choice of reflections for refinement. *R*-factors based on *F*^2^ are statistically about twice as large as those based on *F*, and *R*- factors based on ALL data will be even larger.

### Fractional atomic coordinates and isotropic or equivalent isotropic displacement parameters (Å^2^)

**Table:**

	*x*	*y*	*z*	*U*_iso_*/*U*_eq_	
C1	0.1822 (4)	0.5189 (4)	0.2581 (4)	0.0371 (8)	
C2	0.1565 (4)	0.5725 (4)	0.1253 (4)	0.0397 (9)	
C3	0.2419 (4)	0.5213 (4)	−0.0106 (4)	0.0411 (9)	
C4	0.3642 (5)	0.4250 (4)	−0.0615 (5)	0.0527 (11)	
H4B	0.4128	0.3779	−0.0068	0.063*	
C5	0.3934 (5)	0.4173 (5)	−0.2057 (5)	0.0574 (11)	
H5B	0.4667	0.3614	−0.2667	0.069*	
C6	0.2055 (5)	0.5708 (4)	−0.1327 (4)	0.0435 (9)	
C7	0.0930 (5)	0.6655 (4)	−0.1257 (5)	0.0479 (10)	
H7B	0.0734	0.6951	−0.2083	0.057*	
C8	0.0109 (5)	0.7143 (4)	0.0100 (5)	0.0489 (10)	
H8B	−0.0661	0.7774	0.0196	0.059*	
C9	0.0433 (4)	0.6690 (4)	0.1319 (4)	0.0405 (9)	
H9B	−0.0124	0.7042	0.2212	0.049*	
C10	0.2369 (4)	0.9946 (3)	0.2932 (4)	0.0361 (8)	
C11	0.3995 (4)	0.9972 (4)	0.2597 (4)	0.0369 (8)	
C12	0.4597 (4)	0.9141 (4)	0.3474 (4)	0.0364 (8)	
C13	0.3959 (5)	0.8222 (4)	0.4804 (4)	0.0427 (9)	
H13A	0.2940	0.7979	0.5327	0.051*	
C14	0.5141 (5)	0.7769 (4)	0.5158 (5)	0.0484 (10)	
H14A	0.5046	0.7167	0.5984	0.058*	
C15	0.6195 (4)	0.9196 (4)	0.3068 (4)	0.0400 (9)	
C16	0.7170 (5)	1.0037 (4)	0.1850 (5)	0.0506 (10)	
H16A	0.8210	1.0050	0.1601	0.061*	
C17	0.6541 (5)	1.0853 (5)	0.1024 (5)	0.0531 (11)	
H17A	0.7168	1.1432	0.0209	0.064*	
C18	0.4975 (5)	1.0825 (4)	0.1391 (5)	0.0466 (10)	
H18A	0.4583	1.1389	0.0816	0.056*	
N1	0.1790 (4)	0.5964 (3)	0.3603 (3)	0.0386 (7)	
H1A	0.1685	0.6838	0.3552	0.046*	
N2	0.1950 (4)	0.5168 (3)	0.4730 (4)	0.0446 (8)	
N3	0.2064 (4)	0.3928 (3)	0.4385 (4)	0.0467 (8)	
N4	0.2006 (4)	0.3901 (3)	0.3040 (4)	0.0435 (8)	
N5	0.3005 (4)	0.5030 (4)	−0.2493 (4)	0.0547 (9)	
H5A	0.3015	0.5130	−0.3365	0.066*	
N9	0.1456 (3)	0.8869 (3)	0.3328 (4)	0.0400 (8)	
N8	0.0065 (4)	0.9348 (3)	0.3460 (4)	0.0469 (8)	
N7	0.0106 (4)	1.0667 (3)	0.3174 (4)	0.0467 (8)	
N6	0.1544 (4)	1.1054 (3)	0.2838 (4)	0.0412 (8)	
H6A	0.1880	1.1883	0.2601	0.049*	
N10	0.6481 (4)	0.8330 (3)	0.4119 (4)	0.0470 (8)	
H10A	0.7363	0.8168	0.4121	0.056*	

### Atomic displacement parameters (Å^2^)

**Table:**

	*U*^11^	*U*^22^	*U*^33^	*U*^12^	*U*^13^	*U*^23^
C1	0.040 (2)	0.0331 (18)	0.039 (2)	−0.0061 (15)	−0.0166 (16)	−0.0030 (15)
C2	0.044 (2)	0.0339 (18)	0.045 (2)	−0.0051 (16)	−0.0215 (18)	−0.0025 (15)
C3	0.041 (2)	0.040 (2)	0.043 (2)	−0.0047 (16)	−0.0178 (17)	−0.0036 (16)
C4	0.049 (2)	0.042 (2)	0.069 (3)	0.0052 (18)	−0.025 (2)	−0.011 (2)
C5	0.056 (3)	0.057 (3)	0.052 (3)	0.000 (2)	−0.013 (2)	−0.014 (2)
C6	0.051 (2)	0.048 (2)	0.034 (2)	−0.0138 (18)	−0.0212 (17)	0.0030 (16)
C7	0.054 (2)	0.049 (2)	0.050 (2)	−0.0084 (19)	−0.032 (2)	0.0047 (18)
C8	0.043 (2)	0.042 (2)	0.065 (3)	−0.0011 (17)	−0.027 (2)	−0.0027 (19)
C9	0.038 (2)	0.0358 (19)	0.048 (2)	0.0008 (15)	−0.0181 (17)	−0.0065 (16)
C10	0.043 (2)	0.0288 (17)	0.044 (2)	0.0036 (15)	−0.0243 (17)	−0.0075 (15)
C11	0.0369 (19)	0.0323 (18)	0.044 (2)	0.0016 (15)	−0.0188 (16)	−0.0067 (15)
C12	0.0382 (19)	0.0326 (18)	0.043 (2)	0.0034 (15)	−0.0198 (16)	−0.0081 (15)
C13	0.046 (2)	0.040 (2)	0.043 (2)	0.0027 (16)	−0.0186 (18)	−0.0048 (16)
C14	0.053 (2)	0.050 (2)	0.047 (2)	0.0083 (19)	−0.027 (2)	−0.0031 (18)
C15	0.0348 (19)	0.040 (2)	0.050 (2)	0.0055 (15)	−0.0217 (17)	−0.0128 (17)
C16	0.041 (2)	0.052 (2)	0.058 (3)	−0.0035 (18)	−0.019 (2)	−0.011 (2)
C17	0.050 (2)	0.055 (2)	0.052 (2)	−0.010 (2)	−0.019 (2)	0.0002 (19)
C18	0.051 (2)	0.040 (2)	0.051 (2)	−0.0028 (17)	−0.026 (2)	0.0036 (17)
N1	0.0492 (18)	0.0278 (15)	0.0448 (18)	0.0002 (13)	−0.0255 (15)	−0.0034 (12)
N2	0.059 (2)	0.0448 (19)	0.0367 (17)	−0.0017 (15)	−0.0272 (16)	0.0015 (14)
N3	0.058 (2)	0.0412 (18)	0.049 (2)	0.0004 (15)	−0.0331 (17)	0.0069 (14)
N4	0.051 (2)	0.0360 (17)	0.0468 (19)	0.0025 (14)	−0.0231 (16)	−0.0047 (14)
N5	0.061 (2)	0.063 (2)	0.0390 (19)	−0.0047 (18)	−0.0200 (17)	−0.0027 (16)
N9	0.0388 (17)	0.0334 (16)	0.055 (2)	0.0025 (13)	−0.0259 (15)	−0.0060 (14)
N8	0.0436 (19)	0.0404 (18)	0.065 (2)	0.0011 (14)	−0.0299 (17)	−0.0063 (16)
N7	0.0447 (19)	0.0421 (18)	0.059 (2)	0.0022 (14)	−0.0278 (17)	−0.0018 (15)
N6	0.0442 (18)	0.0301 (15)	0.056 (2)	0.0027 (13)	−0.0278 (16)	−0.0020 (13)
N10	0.0412 (18)	0.053 (2)	0.057 (2)	0.0129 (15)	−0.0305 (17)	−0.0113 (16)

### Geometric parameters (Å, °)

**Table:**

C1—N4	1.326 (5)	C12—C15	1.427 (5)
C1—N1	1.333 (5)	C12—C13	1.430 (5)
C1—C2	1.479 (5)	C13—C14	1.370 (6)
C2—C9	1.404 (5)	C13—H13A	0.9300
C2—C3	1.407 (5)	C14—N10	1.368 (6)
C3—C6	1.428 (5)	C14—H14A	0.9300
C3—C4	1.441 (6)	C15—N10	1.382 (5)
C4—C5	1.357 (6)	C15—C16	1.390 (6)
C4—H4B	0.9300	C16—C17	1.381 (6)
C5—N5	1.369 (6)	C16—H16A	0.9300
C5—H5B	0.9300	C17—C18	1.402 (6)
C6—N5	1.376 (5)	C17—H17A	0.9300
C6—C7	1.386 (6)	C18—H18A	0.9300
C7—C8	1.388 (6)	N1—N2	1.350 (4)
C7—H7B	0.9300	N1—H1A	0.8600
C8—C9	1.396 (6)	N2—N3	1.295 (5)
C8—H8B	0.9300	N3—N4	1.369 (4)
C9—H9B	0.9300	N5—H5A	0.8600
C10—N9	1.323 (5)	N9—N8	1.364 (4)
C10—N6	1.341 (4)	N8—N7	1.295 (4)
C10—C11	1.465 (5)	N7—N6	1.347 (4)
C11—C18	1.392 (5)	N6—H6A	0.8600
C11—C12	1.406 (5)	N10—H10A	0.8600
			
N4—C1—N1	107.9 (3)	C14—C13—C12	106.7 (4)
N4—C1—C2	128.4 (3)	C14—C13—H13A	126.7
N1—C1—C2	123.5 (3)	C12—C13—H13A	126.7
C9—C2—C3	118.4 (4)	N10—C14—C13	110.2 (4)
C9—C2—C1	121.9 (3)	N10—C14—H14A	124.9
C3—C2—C1	119.7 (3)	C13—C14—H14A	124.9
C2—C3—C6	117.1 (4)	N10—C15—C16	130.6 (4)
C2—C3—C4	135.0 (4)	N10—C15—C12	106.8 (3)
C6—C3—C4	107.9 (4)	C16—C15—C12	122.6 (4)
C5—C4—C3	105.7 (4)	C17—C16—C15	117.7 (4)
C5—C4—H4B	127.2	C17—C16—H16A	121.2
C3—C4—H4B	127.2	C15—C16—H16A	121.2
C4—C5—N5	110.8 (4)	C16—C17—C18	121.2 (4)
C4—C5—H5B	124.6	C16—C17—H17A	119.4
N5—C5—H5B	124.6	C18—C17—H17A	119.4
N5—C6—C7	129.9 (4)	C11—C18—C17	121.4 (4)
N5—C6—C3	105.7 (4)	C11—C18—H18A	119.3
C7—C6—C3	124.4 (4)	C17—C18—H18A	119.3
C6—C7—C8	117.1 (4)	C1—N1—N2	109.6 (3)
C6—C7—H7B	121.4	C1—N1—H1A	125.2
C8—C7—H7B	121.4	N2—N1—H1A	125.2
C7—C8—C9	120.3 (4)	N3—N2—N1	105.8 (3)
C7—C8—H8B	119.8	N2—N3—N4	110.8 (3)
C9—C8—H8B	119.8	C1—N4—N3	105.9 (3)
C8—C9—C2	122.7 (4)	C5—N5—C6	109.9 (4)
C8—C9—H9B	118.7	C5—N5—H5A	125.0
C2—C9—H9B	118.7	C6—N5—H5A	125.0
N9—C10—N6	107.4 (3)	C10—N9—N8	106.6 (3)
N9—C10—C11	128.0 (3)	N7—N8—N9	110.3 (3)
N6—C10—C11	124.6 (3)	N8—N7—N6	106.3 (3)
C18—C11—C12	118.8 (3)	C10—N6—N7	109.4 (3)
C18—C11—C10	120.0 (3)	C10—N6—H6A	125.3
C12—C11—C10	121.2 (3)	N7—N6—H6A	125.3
C11—C12—C15	118.3 (3)	C14—N10—C15	109.3 (3)
C11—C12—C13	134.6 (3)	C14—N10—H10A	125.4
C15—C12—C13	107.0 (3)	C15—N10—H10A	125.4
			
N4—C1—C2—C9	133.5 (4)	C12—C13—C14—N10	−1.0 (5)
N1—C1—C2—C9	−41.2 (5)	C11—C12—C15—N10	178.1 (3)
N4—C1—C2—C3	−43.3 (6)	C13—C12—C15—N10	0.4 (4)
N1—C1—C2—C3	142.1 (4)	C11—C12—C15—C16	0.2 (5)
C9—C2—C3—C6	−0.7 (5)	C13—C12—C15—C16	−177.5 (4)
C1—C2—C3—C6	176.2 (3)	N10—C15—C16—C17	−176.8 (4)
C9—C2—C3—C4	179.7 (4)	C12—C15—C16—C17	0.6 (6)
C1—C2—C3—C4	−3.4 (6)	C15—C16—C17—C18	−0.6 (6)
C2—C3—C4—C5	178.1 (4)	C12—C11—C18—C17	1.1 (6)
C6—C3—C4—C5	−1.5 (4)	C10—C11—C18—C17	−178.7 (4)
C3—C4—C5—N5	0.9 (5)	C16—C17—C18—C11	−0.2 (7)
C2—C3—C6—N5	−178.3 (3)	N4—C1—N1—N2	−0.2 (4)
C4—C3—C6—N5	1.4 (4)	C2—C1—N1—N2	175.4 (3)
C2—C3—C6—C7	0.4 (6)	C1—N1—N2—N3	−0.5 (4)
C4—C3—C6—C7	−180.0 (4)	N1—N2—N3—N4	0.9 (4)
N5—C6—C7—C8	178.0 (4)	N1—C1—N4—N3	0.8 (4)
C3—C6—C7—C8	−0.3 (6)	C2—C1—N4—N3	−174.6 (4)
C6—C7—C8—C9	0.5 (6)	N2—N3—N4—C1	−1.1 (4)
C7—C8—C9—C2	−0.9 (6)	C4—C5—N5—C6	−0.1 (5)
C3—C2—C9—C8	1.0 (5)	C7—C6—N5—C5	−179.4 (4)
C1—C2—C9—C8	−175.8 (3)	C3—C6—N5—C5	−0.8 (4)
N9—C10—C11—C18	137.4 (4)	N6—C10—N9—N8	0.5 (4)
N6—C10—C11—C18	−40.4 (5)	C11—C10—N9—N8	−177.6 (4)
N9—C10—C11—C12	−42.3 (6)	C10—N9—N8—N7	−0.6 (4)
N6—C10—C11—C12	139.8 (4)	N9—N8—N7—N6	0.5 (4)
C18—C11—C12—C15	−1.1 (5)	N9—C10—N6—N7	−0.2 (4)
C10—C11—C12—C15	178.7 (3)	C11—C10—N6—N7	178.0 (3)
C18—C11—C12—C13	175.9 (4)	N8—N7—N6—C10	−0.2 (4)
C10—C11—C12—C13	−4.3 (6)	C13—C14—N10—C15	1.3 (5)
C11—C12—C13—C14	−176.9 (4)	C16—C15—N10—C14	176.6 (4)
C15—C12—C13—C14	0.3 (4)	C12—C15—N10—C14	−1.0 (4)

### Hydrogen-bond geometry (Å, °)

**Table:**

*D*—H···*A*	*D*—H	H···*A*	*D*···*A*	*D*—H···*A*
N1—H1A···N9	0.86	2.00	2.858 (4)	177.
N6—H6A···N4^i^	0.86	2.10	2.899 (4)	154.

Symmetry codes:  (i) *x*, *y*+1, *z*.
